# Natriuretic peptide testing for the evaluation of critically ill patients with shock in the intensive care unit: a prospective cohort study

**DOI:** 10.1186/cc4839

**Published:** 2006-02-22

**Authors:** James L Januzzi, Alexander Morss, Roderick Tung, Richard Pino, Michael A Fifer, B Taylor Thompson, Elizabeth Lee-Lewandrowski

**Affiliations:** 1 Division of Cardiology*, Massachusetts General Hospital, Boston, Massachusetts, USA; 2 Department of Medicine†, Massachusetts General Hospital, Boston, Massachusetts, USA; 3 Department of Anesthesia and Critical Care‡, Massachusetts General Hospital, Boston, Massachusetts, USA; 4 Department of Pulmonary/Critical Care, Massachusetts General Hospital, Boston, Massachusetts, USA; 5 Department of Laboratory Medicine, Massachusetts General Hospital, Boston, Massachusetts, USA

## Abstract

**Introduction:**

Amino-terminal pro-brain natriuretic peptide (NT-proBNP) is useful in evaluating heart failure, but its role in evaluating patients with shock in the intensive care unit (ICU) is not clear.

**Method:**

Forty-nine consecutive patients in four different ICUs with shock of various types and with an indication for pulmonary artery catheter placement were evaluated. Analyses for NT-proBNP were performed on blood obtained at the time of catheter placement and results were correlated with pulmonary artery catheter findings. Logistic regression identified independent predictors of mortality.

**Results:**

A wide range of NT-proBNP levels were observed (106 to >35,000 pg/ml). There was no difference in median NT-proBNP levels between patients with a cardiac and those with a noncardiac origin to their shock (3,046 pg/ml versus 2,959 pg/ml; *P *= 0.80), but an NT-proBNP value below 1,200 pg/ml had a negative predictive value of 92% for cardiogenic shock. NT-proBNP levels did not correlate with filling pressures or hemodynamics (findings not significant). NT-proBNP concentrations were higher in patients who died in the ICU (11,859 versus 2,534 pg/ml; *P *= 0.03), and the mortality rate of patients in the highest log-quartile of NT-proBNP (66.7%) was significantly higher than those in other log-quartiles (*P *< 0.001); NT-proBNP independently predicted ICU mortality (odds ratio 14.8, 95% confidence interval 1.8–125.2; *P *= 0.013), and was superior to Acute Physiology and Chronic Health Evaluation II score and brain natriuretic peptide in this regard.

**Conclusion:**

Elevated levels of NT-proBNP do not necessarily correlate with high filling pressures among patients with ICU shock, but marked elevation in NT-proBNP is strongly associated with ICU death. Low NT-proBNP values in patients with ICU shock identifed those at lower risk for death, and may be useful in excluding the need for pulmonary artery catheter placement in such patients.

## Introduction

Patients with shock in the intensive care unit (ICU) setting are at high risk for morbidity and mortality, and optimal strategies to evaluate and manage ICU patients with shock are needed. One method to evaluate patients with ICU shock includes invasive hemodynamic monitoring with pulmonary artery catheter (PAC) placement. Such invasive monitoring, although widely employed, has been shown to be associated only equivocally with improved outcomes in the ICU setting [1] and, in fact, it may be associated with a null effect on outcomes [2-4] or with increased hazard in certain individuals [1,5]. Accordingly, a noninvasive method for estimating cardiac filling pressures and hemodynamics in ICU patients with shock would be desirable.

Biomarker measurement has been used in the optimal evaluation and management of multiple disease states, ranging from endocrinologic states such as diabetes or thyroid diseases to cardiovascular diseases such as acute coronary syndromes and heart failure. However, the role of biomarkers in the evaluation of patients in the ICU setting remains largely unexplored. Because hemodynamic monitoring is a frequent theme in the evaluation of patients in the ICU setting, there may be logic behind evaluating ICU patients by testing for B-type (brain) natriuretic peptide (BNP) or its amino-terminal pro-fragment NT-proBNP. These markers are useful in diagnosing or excluding heart failure [6,7] as well as in estimating prognosis [8] and for managing patients with heart failure [9]. In patients with relatively uncomplicated heart failure, concentrations of natriuretic peptides correlate to a degree with cardiac filling pressures and hemodynamics, particularly within the same individual [10,11]. The implication is that measurement of natriuretic peptides might be useful in that they may allow invasive monitoring to be avoided in some patients with heart failure.

Given their value in evaluating and managing patients with heart failure, the natriuretic peptide class of biomarkers might be useful as a surrogate for PAC placement in patients with undefined hemodynamics, in whom PAC placement might otherwise be contemplated. In ICU patients with undifferentiated shock, we recently demonstrated that BNP concentrations are frequently elevated but unrelated to cardiac filling pressures or hemodynamics [12]. Despite the lack of association between BNP and cardiac parameters, BNP strongly predicted ICU mortality. Less is known about NT-proBNP levels in ICU shock patients [13] and whether any additive information might be gained from using NT-proBNP rather than BNP. Accordingly, we performed the present analysis to evaluate the behavior of NT-proBNP in patients with shock in the ICU, to examine the relationship between NT-proBNP and cardiac hemodynamics/filling pressures, to evaluate the utility of NT-proBNP to predict ICU death in patients with shock, and to examine the role of NT-proBNP with other biomarkers in estimating prognosis in the ICU.

## Materials and methods

### Study design

This study was approved by the institutional review board and was conducted in the surgical, cardiac, and medical ICUs at the Massachusetts General Hospital. The design of the predecessor BNP study was previously reported [12]. The present study examined patients from the same cohort, with two patients in the previous data set removed because of lack of NT-proBNP data and two patients without BNP data in the prior analysis added, leaving the same number of patients for the present analysis. In brief, the 49 patients were prospectively enrolled. They met both of the following inclusion criteria: hypotension, defined as a systolic blood pressure below 100 mmHg with end-organ dysfunction or need for vasopressor or inotropic agents (including norepinephrine [noradrenaline], phenylephrine, vasopressin, dopamine, dobutamine, or milrinone); and intention by the clinicians managing the patient to place a PAC for diagnosis or monitoring as part of standard care.

For each patient, 5 ml blood was collected into a tube containing EDTA at the time of PAC placement. Samples were processed and analyzed for BNP concentrations (Triage; Biosite, LaJolla, CA, USA). Following the original study, frozen samples from each patient were thawed and NT-proBNP concentrations were measured using a commercially available immunoassay (Elecsys proBNP; Roche Diagnostics, Indianapolis, IN, USA). The patients, researchers and ICU physicians were blinded to natriuretic peptide findings.

Clinical characteristics, including demographics, past medical history, presenting syndrome, PAC variables (including filling pressures and hemodynamics), and subsequent hospital course, were recorded.

Cardiac hemodynamics (including cardiac index) were calculated using the thermodilution technique and pulmonary capillary wedge pressures (PCWPs) were measured at end expiration. Acute Physiology and Chronic Health Evaluation (APACHE) II scores were calculated using data available from the 24-hour period at the time of enrollment.

Patients were classified on a binary system as having shock of cardiac or noncardiac origin. The former group ('cardiac origin') included patients with symptoms/signs of shock following an acute myocardial infarction with or without acute destabilized heart failure. In addition, we further considered a subgroup of patients within the category of 'cardiac origin' of shock, namely those with cardiogenic shock, defined as having suffered a cardiac event associated with the combination of both cardiac index below 2.2 l/min per m^2 ^and PCWP above 18 mmHg. The 'noncardiac' group included those with distributive, hypovolemic, and mixed shock etiologies.

The primary end-points for this study were similar to those in the original report [12], with substitution of NT-proBNP levels for BNP levels for correlation with PCWP and cardiac index. An analysis to determine the negative predictive value of NT-proBNP in order to rule out cardiogenic shock was performed, and the utility of NT-proBNP in predicting ICU death was examined, including a comparison of NT-proBNP results with the results of BNP testing as well as with APACHE II scoring [14].

### Statistical analysis

NT-proBNP levels were log-transformed to achieve normality. Correlations between NT-proBNP values and PCWP and between NT-proBNP values and cardiac index were performed by bivariate analyses with Spearman correlation. Comparisons of median NT-proBNP levels between survivors and those who died were made by nonparametric testing.

In order to analyze the prognostic influence of NT-proBNP in the ICU, receiver operating characteristic analyses for death as a function of NT-proBNP concentration were performed, using Analyze-it software (Analyze-it, Leeds, UK). Patients were categorized into quartiles using log-transformed NT-proBNP values. Multivariable analysis utilizing stepwise logistic regression was used to identify independent predictors of death. Factors included in the multivariable model included age, sex, diagnosis of cardiogenic shock, use of mechanical ventilation, cardiac troponin T concentration, renal function as indicated by serum creatinine, blood pH, BNP concentration, and APACHE II score. Logistic regression analysis used stepping with verification of goodness-of-fit with the Hosmer-Lemeshow test. Odds ratios were generated and expressed with 95% confidence intervals. All *P *values are two-sided, and *P *< 0.05 was considered statistically significant. All statistical analyses were performed using SPSS software (SPSS Institute Inc., Chicago, IL, USA).

## Results

The baseline characteristics of the study group are shown in Table 1. Approximately half of the patients had shock of cardiac origin, whereas the remaining patients had shock of different etiologies. Seven patients had cardiogenic shock, as defined above.

### NT-proBNP levels and hemodynamics

The median (interquartile range) NT-proBNP for all patients was 3,046 (1,074–16,068) pg/ml; a wide range of NT-proBNP values was documented (106 to >35,000 pg/ml). Seven patients (14%) had NT-proBNP values below the threshold for diagnosis of heart failure, as defined previously [6]. Although patients with a cardiac source to their shock (including the seven with cardiogenic shock) had significantly higher median (interquartile range) NT-proBNP concentrations (4,390 [2,444–17,951] pg/ml) than did those with noncardiac sources of shock (2,148 [870–11,585] pg/ml; *P *= 0.02 for the difference), there was no statistically significant difference in median NT-proBNP levels between patients with cardiogenic (as defined above) and the rest of the patients in the study (3,046 pg/ml versus 3,380 pg/ml; *P *= 0.88). Notably, an NT-proBNP of below 1,200 pg/ml had a negative predictive value of 92% for ruling out the hemodynamic indices and filling pressures consistent with cardiogenic shock.

**Figure 1:**
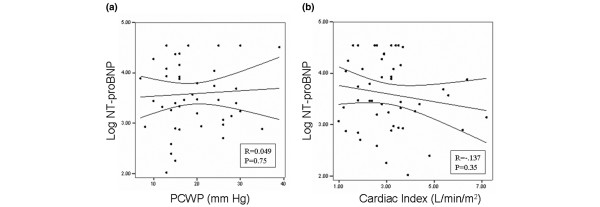
Relationship between NT-proBNP, and PCWP and cardiac index. Shown are linear regression analyses examining the relationship between log-transformed amino-terminal pro-brain natriuretic peptide (NT-proBNP) concentrations and **(a) **pulmonary capillary wedge pressure (PCWP), and **(b) **cardiac index.

Similar to the data reported for BNP in this group, we found no correlation between NT-proBNP concentrations and either PCWP (*r *= 0.05, *P *= 0.75) or cardiac index (*r *= -0.137, *P *= 0.35) at the time of PAC placement (Figure 1).

### Mortality

**Figure 2:**
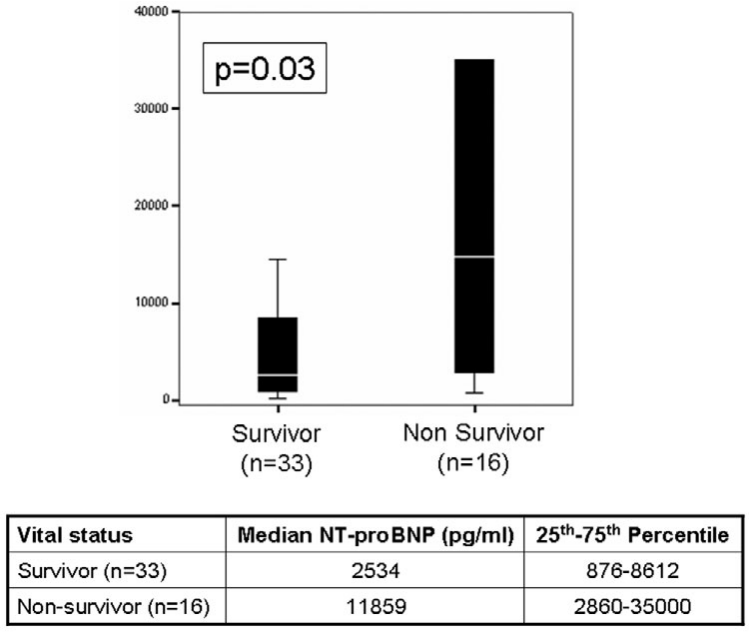
Concentrations of NT-proBNP as a function of survival versus death in the ICU. The line refers to the median, whereas the boxes refer to the interquartile ranges. The whiskers represent the 5th and 95th centiles, respectively. ICU, intensive care unit; NT-proBNP, amino-terminal pro-brain natriuretic peptide.

Sixteen patients (33%) died during the course of the study. The median NT-proBNP levels in patients who died were significantly higher than in those who survived (11,859 pg/ml versus 2,534 pg/ml; *P *= 0.03; Figure 2). Receiver operating characteristic analyses examining NT-proBNP for mortality prediction demonstrated an area under the curve of 0.72 (*P *= 0.004); notably, an NT-proBNP level below 1000 pg/ml had a negative predictive value of 98% for ICU death.

**Figure 3:**
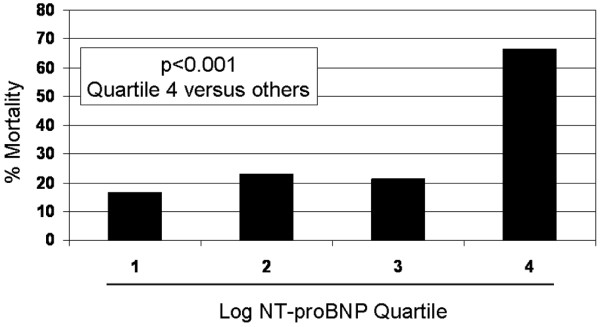
Relationship between NT-proBNP log-quartiles and ICU death. ICU, intensive care unit; NT-proBNP, amino-terminal pro-brain natriuretic peptide.

Figure 3 demonstrates the relationship between NT-proBNP concentrations and mortality rates. The lowest rate of ICU mortality (16.7%) was in those patients with NT-proBNP concentrations in the lowest log-quartile, whereas those in the highest log-quartile had the highest rates of death (67%); the mortality risks in the second and third log-quartiles (at 23% and 21%, respectively) were comparable to each other and slightly higher than that in the lowest log-quartile. The difference between the rate of mortality in the highest log-quartile relative to all other log-quartiles was statistically significant (*P *< 0.001).

Several factors were found by multivariate analyses to be significant predictors of death in the ICU (Table 2), including blood pH ≤ 7.15 and serum creatinine ≥ 1.8 mg/dl. Strong trends were noted for history of prior heart failure and mechanical ventilation use. However, an NT-proBNP in the highest log-quartile (corresponding to an NT-proBNP ≥ 17,568 pg/ml) was the factor most strongly predictive of death (odds ratio 14.8, 95% confidence interval 1.8–125.2; *P *= 0.013). Although in univariate analyses BNP concentrations in the highest log-quartile (odds ratio 4.9, 95% confidence interval 1.2–10.4; *P *= 0.04) and APACHE II scores ≥ 25 (odds ratio 2.0, 95% confidence interval 1.1–11.5; *P *= 0.05) were both significant predictors of death, in multivariate models that included NT-proBNP neither BNP nor APACHE II scores were significantly associated with ICU death.

## Discussion

In patients with ICU shock NT-proBNP concentrations did not correlate with cardiac hemodynamics or with filling pressures, but NT-proBNP levels were considerably higher among those patients who died in the ICU and represented the strongest predictor of death in the ICU. Conversely, low NT-proBNP levels identified shock patients at lower risk for death. Notably, in a multivariate model identifying independent predictors of death in the ICU, NT-proBNP was superior to BNP and APACHE II scoring, both of which were previously identified as being useful for prognostication in patients with ICU shock.

Regarding the dissociation between natriuretic peptide concentrations and filling pressures, we previously demonstrated a similar lack of association between BNP and cardiac parameters in ICU shock [12], and a recent report from Forfia and colleagues [15] also demonstrated the lack of association between natriuretic peptide concentrations and PCWP in shock patients. The lack of association between natriuretic peptides and cardiac filling pressures or hemodynamics in ICU shock reflects structural heart disease – either pre-existing or developing acutely – related to endotoxemia, cytokines, or catecholamine infusions used in the majority of our patients. Each may be accompanied by subtle myocardial abnormalities in patients with shock. These factors may also stimulate the release of natriuretic peptides or a fall in cardiac index [16-18] in the absence of elevated filling pressures. In addition, the independent relationship between natriuretic peptides and renal function (frequently abnormal in the setting of the multiorgan system dysfunction that characterizes shock) also potentially suggests that the increased levels of NT-proBNP in patients with poorer prognosis reflected reduced clearance of the marker.

In the present study, although an elevated NT-proBNP had poor predictive value for identifying elevated filling pressures, an NT-proBNP concentration below 1,200 pg/ml had a negative predictive value of 92% for the combination of cardiac index below 2.2 l/min per m^2 ^and PCWP above 18 mmHg – the classical definition of cardiogenic shock. In this setting of an NT-proBNP below 1,200 pg/ml (a situation noted in approximately 29% of patients), a clinician concerned about the presence of elevated cardiac filling pressures or abnormal hemodynamic parameters, and otherwise inclined to place a PAC, might consider conservative management without the potential risk associated with PAC placement. That such patients with lower NT-proBNP concentrations were also generally at lower risk further underscores the potential utility for natriuretic peptide 'screening' in the hope of potentially avoiding PAC placement, because such lower risk patients are those in whom an unacceptable risk/benefit ratio from PAC placement has been described [1].

The severity of the processes that underlie release of natriuretic peptide appear to parallel that of risk for mortality; this risk is contributed to, but independent of, renal function, because NT-proBNP was equally important for risk estimation in critically ill ICU patients even in the presence of abnormal serum creatinine levels. Notably, although renal function, which is known to be a potent measure of prognosis in the ICU setting [19,20], was an independent predictor of death in our study, the risk associated with abnormal renal function was additive to that of NT-proBNP. Thus, the elevated levels of NT-proBNP and the associated increased risk for mortality in this setting could not entirely be accounted for by reduced clearance of the marker.

Estimating prognosis in the ICU may be challenging, and in a patient population with shock in the ICU – although high mortality rates are expected – a simple, reproducible, and especially reliable method for stratifying risk for death is welcome. Established methods for estimating risk include the use of risk scoring systems, such as the APACHE II score [14]. Because of complexity, however, the APACHE II score has not gained widespread use in routine ICU patient evaluation. In our analysis, NT-proBNP concentrations as a single measure were superior to APACHE II scores for predicting mortality, in a manner similar to that of BNP from prior reports [12]. This finding is consistent with previous reports from our group [12] and others [13,21] suggesting the utility of natriuretic peptide testing for prognosis estimation, even in noncardiogenic shock states.

In the present study, we compared the utility of both natriuretic peptide markers for their ability to predict death. We demonstrated – in the first head-to-head analysis of NT-proBNP and BNP testing in the ICU – that, in the presence of NT-proBNP results, BNP results added no further prognostic information and was no longer a significant predictor of death. These findings to not necessarily contradict those from prior reports suggesting the value of BNP in prognosticating ICU shock [12], but they suggest a potential superiority of NT-proBNP over BNP for this indication.

Finally, it is necessary to emphasize that those patients below the fourth log-quartile for NT-proBNP did not have a benign outcome, with a mortality rate at or around 20% in each log-quartile below the fourth. Such patients could be further substratified using the results of serum creatinine and blood pH, such that those with an NT-proBNP below the fourth log quartile, serum creatinine <1.8 mg/ml, and a blood pH ≥ 7.15 have the lowest risk for death (<10%) whereas those with all three risk factors are at greatest risk for ICU death (80%). Although data exist supporting the concept that individual biomarkers may predict ICU mortality, including natriuretic peptides [12,21], serum creatinine [19,20] and blood pH [22-24], to our knowledge our study is the first to describe the independent relationship(s) between these markers in a prognostic model for ICU death.

Limitations of the present study include the fact that PAC placement was done at the discretion of the managing physician rather than as part of a protocol-based decision-making process. However, PAC placement in shock is not widely recommended unless it is clinically indicated using the same decision-making process as was employed by our clinicians [25], and so the use of PAC in the study probably represents that in a 'real life' situation. Also, it has been suggested that serial intrapatient measurements of natriuretic peptide might more adequately correlate with filling pressures, rather than single measurements [10], and so serial measurement of NT-proBNP or BNP might still be useful in estimating filling pressures. We argue that observations supporting the value of serial testing for estimating hemodynamics are largely based on experience in patients with destabilized heart failure, rather than in patients who are critically ill with shock, and thus remain speculative. We further point out that the levels of natriuretic peptide that we and others have reported in patients with ICU shock are often of a magnitude typically seen with very severe, life-threatening acute destabilized heart failure [12,13,15] but without elevated filling pressure. Thus, in the setting of critical illness we suggest that release of NT-proBNP or BNP probably represents release from the myocardium in response to signals other than myocyte stretch, which is the usual paradigm for NT-proBNP or BNP release [16].

## Conclusion

High concentrations of NT-proBNP should not be used to estimate high cardiac filling pressures or impaired hemodynamics in ICU patients with undefined shock; however, low concentrations of NT-proBNP in patients with shock confidently excludes elevated filling pressures/low cardiac index, and identifies a low-risk patient in whom placement of a PAC might not be expected to be useful. In light of the recent call for a more thoughtful approach when considering which patients are eligible for PAC placement [25,26], the use of natriuretic peptides to reduce the number of patients in whom this potentially risky procedure is performed is a reasonable consideration. Further studies using larger cohorts of ICU shock patients should elucidate the diagnostic and prognostic role of natriuretic peptides in this complex patient population.

## Key messages

• 	In random patients with shock of various types in the ICU setting, elevated NT-proBNP concentrations were common and not necessarily related to high filling pressures or low cardiac output.

• 	Elevated levels of NT-proBNP were strongly associated with risk for death in the ICU, and were a stronger predictor of death than APACHE II scoring.

• 	A low level of NT-proBNP identified a low risk patient in whom lower filling pressures were expected. Because these lower risk patients are less likely to benefit from PAC placement, a low NT-proBNP value in ICU shock would allow clinicians to reconsider placement of a PAC.

## Abbreviations

APACHE = Acute Physiology and Chronic Health Evaluation; BNP = brain natriuretic peptide; ICU = intensive care unit; NT-proBNP = amino-terminal pro-brain natriuretic peptide; PAC = pulmonary artery catheter; PCWP = pulmonary capillary wedge pressure.

## Competing interests

Dr Januzzi reports having received grant support, consulting fees, and speaking fees from Roche Diagnostics, Inc.

## Authors' contributions

JLJ conceived the study, procured study funds, performed data analysis, and drafted the manuscript. AM collected data, performed data analysis, and drafted the manuscript. RT conceived the study, collected data, and drafted the manuscript. RP, MAF, and BTT assisted in patient recruitment, and contributed to the drafting of the manuscript. ELL ran the NT-proBNP assays and interpreted data. All authors read and approved the final manuscript.

## Figures and Tables

**Table 1:** Baseline characteristics of the study population

Characteristic	Value
Age (years)	67.8 ± 13.8
Male sex	69%
Prior heart failure	47%
APACHE II score	21.8 ± 6.9
Hemodynamics	
Cardiac index (l/min per m^2^)	3.1 ± 1.4
PCWP (mmHg)	19.2 ± 7.1
Shock origin	
Cardiac^a^	57%
Septic/SIRS	43%
Mechanical ventilation	78%
Serum creatinine (mg/dl)	1.80 (1.05–2.25)
Cardiac troponin T (ng/ml)	0.19 (0.05–1.0)
Blood pH^b^	7.33 ± 2.3
BNP (pg/ml)	482 (194–1340)
NT-proBNP (pg/ml)	3046 (1074–16068)

In total, 49 patients were included. Values are expressed as mean ± standard deviation, percentage, or median (25th–75th centile). ^a^Seven patieints had hemodynamics consistent with cardiogenic shock, as defined in the text. ^b^Closest measurement to time of pulmonary artery catheter placement. APACHE, Acute Physiology and Chronic Health Evaluation; BNP, brain natriuretic peptide; NT-proBNP, amino-terminal pro-brain natriuretic peptide; PCWP, pulmonary capillary wedge pressure; SIRS, systemic inflammatory response syndrome.

**Table 2:** Results of multivariate logistic regression analysis for predictors of intensive care unit death

Predictor	OR (95% CI)	*P*
NT-proBNP in highest log quartile	14.8 (1.8–125.2)	0.013
Blood pH ≤ 7.15	12.6 (1.5–155.7)	0.04
Creatinine above median (1.8 mg/dl)	0.089 (0.012–0.681)	0.02
Mechanical ventilation	10.8 (0.70–165.8)	0.09
History of heart failure	6.9 (0.84–56.3)	0.07
APACHE II score ≥ 25	5.6 (0.29–56.7)	0.44
Cardiac troponin T in highest quartile	3.7 (0.32–43.5)	0.29
Male sex	1.9 (0.4–6.7)	0.885
BNP in highest log quartile	1.1 (0.19–6.1)	0.92
Age	0.985 (0.91–1.05)	0.63
Cardiogenic shock	0.54 (0.04–7.1)	0.63

APACHE, Acute Physiology and Chronic Health Evaluation; BNP, brain natriuretic peptide; CI, confidence interval; NT-proBNP, amino-terminal pro-brain natriuretic peptide; OR, odds ratio.
